# Vitamin D status, metabolic determinants, and association with MASLD: retrospective health-screening cohort mediation analysis

**DOI:** 10.3389/fnut.2026.1922441

**Published:** 2026-09-03

**Authors:** Hui He, Yanfang Xie, Rui Peng, Xiao Pan, Han Xue, Meiyan Chen, Dong Du, Qian Ge, Yan Wu, Mingfei Wang, Yuanxiang Wang, Qingshan Geng, Weiqing Wu

**Affiliations:** 1 Department of Health Management, Shenzhen People’s Hospital, The First Affiliated Hospital, Southern University of Science and Technology, The Second Clinical Medical College, Jinan University, Shenzhen, Guangdong, China; 2 Department of Thoracic Surgery, Shenzhen People’s Hospital, The First Affiliated Hospital, Southern University of Science and Technology, The Second Clinical Medical College, Jinan University, Shenzhen, Guangdong, China; 3 Department of Clinical Nutrition, Shenzhen People’s Hospital, The First Affiliated Hospital, Southern University of Science and Technology, The Second Clinical Medical College, Jinan University, Shenzhen, Guangdong, China; 4 Department of Endocrinology, Shenzhen People’s Hospital, The First Affiliated Hospital, Southern University of Science and Technology, The Second Clinical Medical College, Jinan University, Shenzhen, Guangdong, China; 5 Headquarters of Shenzhen Nanshan Medical Group, Shenzhen, Guangdong, China; 6 Department of Geriatrics, Shenzhen People’s Hospital, The First Affiliated Hospital, Southern University of Science and Technology, The Second Clinical Medical College, Jinan University, Shenzhen, Guangdong, China

**Keywords:** 25-hydroxyvitamin D, hepatic steatosis, lipid metabolism, mediation analysis, metabolic confounding

## Abstract

**Background:**

The relationship between vitamin D and metabolic dysfunction–associated steatotic liver disease (MASLD) remains controversial due to confounding by metabolic risk factors. We aimed to evaluate the association between vitamin D status and MASLD and examine the extent to which metabolic factors statistically contribute to this association.

**Methods:**

In this large retrospective cross-sectional health-screening cohort study, 35,468 adults (mean age 46.3 years [SD 11.8]) undergoing routine health examinations were included. Serum 25-hydroxyvitamin D [25(OH)D] levels were analysed in relation to ultrasonography-defined MASLD. Multivariable logistic regression, partial correlation, and mediation analyses were used to assess independent associations and estimate the proportion of the observed association statistically explained by metabolic factors.

**Results:**

Mean 25(OH)D concentration was 65.4 nmol/L (SD 26.4), and MASLD prevalence was 36.1%. Higher vitamin D levels were associated with lower odds of MASLD after adjustment for age and sex (OR 0.90 [95% CI 0.89–0.91]) and remained significant after additional adjustment for metabolic factors (OR 0.93 [0.92–0.95]). In contrast, associations between vitamin D and most metabolic traits were attenuated after adjustment, indicating non-independent relationships. Mediation analysis showed that 37.6% of the observed association between vitamin D and MASLD was statistically explained by metabolic factors, predominantly triglycerides (39.2%), HDL cholesterol (14.7%), and BMI (13.0%), whereas the remaining proportion was not explained by the measured mediators included in the model. The inverse association between vitamin D and MASLD was consistent across age and sex subgroups.

**Conclusion:**

Vitamin D is independently associated with lower odds of MASLD beyond traditional metabolic factors. While metabolic factors contribute to part of the observed association, the remaining association was not explained by measured metabolic factors and should not be interpreted as evidence of a causal biological pathway. These findings support further investigation of vitamin D status as an associated marker of metabolic liver health and highlight the need for prospective studies evaluating its clinical relevance.

## 1 Introduction

Non-alcoholic fatty liver disease, recently re-termed metabolic dysfunction–associated steatotic liver disease, has emerged as the most prevalent chronic liver disorder worldwide, affecting approximately 29.8% of the global adult population and over 30% of individuals in Asia, with rapidly rising incidence in urban populations (1, 2). MASLD is not only a hepatic condition but a multisystem disease strongly linked to insulin resistance, dyslipidaemia, obesity, and cardiovascular morbidity, and it is projected to become the leading indication for liver transplantation in the coming decades (3–5). Despite its growing burden, effective pharmacological therapies remain limited, underscoring the need to identify modifiable risk factors and mechanistic pathways that could inform prevention and early intervention strategies. Vitamin D, a secosteroid hormone with established roles in calcium homeostasis and bone health, has increasingly been implicated in regulator of energy metabolism, immune function, and inflammatory processes (6–8). Epidemiological studies indicate that vitamin D deficiency remains highly prevalent worldwide, with approximately half of the global population having serum 25-hydroxyvitamin D concentrations below commonly used deficiency thresholds, and a substantially higher proportion exhibiting vitamin D insufficiency (9). The prevalence is particularly notable in Asian populations; in China, recent large-scale analyses have reported that approximately 29.9% of individuals are vitamin D deficient and more than 60% have vitamin D insufficiency, highlighting a significant public health concern (10, 11). Vitamin D status is shaped by multiple interacting determinants, including latitude, sunlight exposure, dietary intake, adiposity, physical activity, and socioeconomic conditions. These upstream factors influence not only circulating 25(OH)D concentrations but also broader metabolic health. Understanding how these determinants contribute to downstream metabolic diseases is essential for designing effective public health interventions, including targeted supplementation, food fortification, and lifestyle modification strategies (12). Recent studies demonstrate that the vitamin D receptor (VDR) signaling suppresses hepatic lipogenesis via downregulation of sterol regulatory element-binding protein-1c (SREBP-1c), attenuates pro-inflammatory cytokine pathways including tumor necrosis factor-*α* (TNF-α) and interleukin-6 (IL-6), and inhibits hepatic stellate cell activation, thereby providing biological plausibility for potential involvement of vitamin D-related pathways in MASLD development (13, 14). Observational studies have consistently reported an inverse association between circulating 25-hydroxyvitaminD [25(OH)D] levels and MASLD prevalence (15). Meta-analyses indicate that vitamin D deficiency is associated with an approximately 1.3–1.5-fold increased risk of MASLD, with lower circulating 25(OH)D levels also correlating with greater histological severity (16). However, the interpretation of these associations remains challenging. A major limitation of the existing literature is inadequate adjustment for key metabolic confounders including adiposity, triglycerides, and insulin resistance which are strongly interrelated with both circulating 25(OH)D levels and MASLD risk. Given this overlap, the observed associations may reflect underlying metabolic dysfunction rather than a direct effect of vitamin D. Consequently, it remains uncertain whether vitamin D represents an independent determinant of hepatic steatosis or primarily serves as a biomarker of metabolic health.

Furthermore, few studies have systematically evaluated the extent to which metabolic factors statistically contribute to the observed association between vitamin D status and MASLD. In particular, the extent to which the observed association is mediated through intermediate metabolic traits such as dyslipidaemia and adiposity versus reflecting an association independent of these measured metabolic factors has not been rigorously quantified. in large observational cohort (17, 18). This represents a critical knowledge gap, as characterizing these associations is important for understanding the potential relevance of vitamin D status in metabolic liver health. In this context, we conducted a health-screening cohort analysis to comprehensively evaluate the relationship between circulating 25-hydroxyvitamin D [25(OH)D] levels and MASLD. Using multivariable modeling and mediation analysis of observed associations, we sought to determine whether vitamin D remains associated with MASLD after adjustment for established metabolic factors and to estimate the proportion of this association statistically explained by measured metabolic variables. In addition, we examined the consistency of these associations across demographic subgroups to assess their robustness. Together, this approach provides a mechanistically informed framework to better define the role of vitamin D in MASLD pathogenesis and to clarify whether the observed association extends beyond traditional metabolic factors.

## 2 Methods

This study was conducted in accordance with the principles of the Declaration of Helsinki. The study protocol was reviewed and approved by the institutional ethics committee of Shenzhen Peoples Hospital and communicated through letter no. LL-KY-2025263-02. Given the retrospective and anonymised nature of the health examination data, the requirement for informed consent was waived by the ethics committee. All data were de-identified prior to analysis to ensure participant confidentiality.

### 2.1 Study design and population

This retrospective cross-sectional health-screening cohort study was conducted at Shenzhen People’s Hospital between January 2022 and December 2025. Data from 35,468 adults [mean age 46.3 years (SD 11.8)] who underwent routine clinical and biochemical assessments, including serum 25-hydroxyvitamin D [25(OH)D] measurement and abdominal ultrasonography, were included in the analysis. Serum 25(OH)D concentrations were measured using magnetic particle chemiluminescence method (Autobio, Zhengzhou, China) according to the manufacturer’s instructions. Results were reported in nmol/L. Internal and external quality-control procedures were performed according to laboratory standards, with intra-assay and inter-assay coefficients of variation of 3.0 and 3.6%, respectively. Participants were included if they had available data on vitamin D status and at least one metabolic parameter. Individuals with missing vitamin D measurements were excluded. Given the observational nature of the dataset, no intervention was performed. The study population reflects a real-world cohort undergoing standardized health screening, providing a representative sample for evaluating metabolic and hepatic conditions.

### 2.2 Data collection and variable definitions

Demographic, anthropometric, biochemical, and imaging data were extracted from routine health examination records. Continuous variables included age, body mass index (BMI), waist circumference, fasting plasma glucose (FPG), glycated hemoglobin (HbA1c), lipid profile [triglycerides (TG), total cholesterol (TC), high-density lipoprotein (HDL), low-density lipoprotein (LDL)], uric acid (UA), and liver enzymes (ALT, AST). Serum 25-hydroxyvitamin D [25(OH)D] concentrations were measured using standardized laboratory methods and analysed both as a continuous variable (per 10 nmol/L increase) and as categorical variables. For clinical interpretation, vitamin D status was classified according to commonly used thresholds as deficient (<50 nmol/L), insufficient (50–75 nmol/L), and sufficient (>75 nmol/L). In addition, quartile-based categorization was used for dose–response analyses (Q1: 5.9–47.4 nmol/L; Q2: 47.4–60.9 nmol/L; Q3: 60.9–78.1 nmol/L; Q4: ≥78.1 nmol/L). MASLD was identified based on abdominal ultrasonography performed during routine health examinations and interpreted by experienced radiologists according to standardized institutional criteria. Ultrasound examinations were performed using standardized abdominal imaging protocols. Interobserver agreement was not assessed because this was a retrospective analysis of routinely acquired clinical imaging data. Ultrasonographic diagnosis was based on increased hepatic echogenicity relative to the renal cortex, blurring of intrahepatic vascular margins, and deep attenuation of ultrasound signals, in the absence of other structural abnormalities. Abdominal ultrasonography was available for a subset of participants undergoing routine health examinations. MASLD prevalence analyses were therefore restricted to participants with available ultrasound assessments.

### 2.3 Data preprocessing and feature selection

Variables with more than 50% missing data were excluded to minimize potential bias and improve analytical robustness. These variables included blood pressure (87% missing), thyroid function indices (approximately 70% missing), calcium measurements (99% missing), *γ*-glutamyl transferase, and alkaline phosphatase. The missingness of these variables primarily reflected routine health examination practices, as certain laboratory tests were selectively performed according to clinical requirements rather than being uniformly assessed in all participants. Variables with lower proportions of missing data, including triglycerides (28% missing), were retained for analysis when sufficient sample size was available. Because the missing data mechanism could not be definitively classified as missing completely at random (MCAR) or missing at random (MAR), complete-case analyses were performed for variables included in each statistical model. The potential influence of missing data and selection bias was considered as a limitation of this study. Continuous variables with ≥50% completeness and binary variables with a prevalence ≥1% were retained for analysis. Normality of continuous variables was assessed using the Shapiro–Wilk test; however, given the large sample size, distributional characteristics and robustness of rank-based methods were considered when selecting statistical approaches.

### 2.4 Statistical analysis

Continuous variables were summarized as mean (SD) and categorical variables as counts (percentages). Given the large sample size, formal normality testing was interpreted cautiously because small deviations from normality may yield statistically significant results. Statistical methods were selected based on distributional characteristics, graphical assessment, and robustness considerations. Rank-based methods were applied for skewed continuous variables where appropriate. Between-group comparisons were performed using the Mann–Whitney U test, and effect sizes were quantified using Cohen’s d. Spearman correlation coefficients were calculated to assess associations between vitamin D and metabolic variables. To address confounding, partial Spearman correlations were performed adjusting for age and sex. Multivariable linear regression models were constructed with serum 25(OH)D as the dependent variable. Models were sequentially adjusted for demographic and metabolic factors, and standardized coefficients (*β*) were used to assess relative importance. Robust (HC3) standard errors were applied. Logistic regression models were used to evaluate the association between serum 25(OH)D concentrations and MASLD status. MASLD was coded as a binary outcome (0 = absence, 1 = presence). Serum 25(OH)D was analysed as a continuous variable (per 10 nmol/L increase) and as quartiles (Q1–Q4). Model 1 was adjusted for age and sex, and Model 2 additionally included BMI, triglycerides, HDL cholesterol, fasting plasma glucose, and uric acid. Odds ratios (ORs) and 95% confidence intervals (CIs) were reported. Dose–response relationships were examined across vitamin D quartiles with adjustment for age and sex. Subgroup analyses stratified by age and sex were performed to assess effect consistency. Mediation analyses were performed using the R package mediation (version 4.5.3) with 5,000 bootstrap simulations. The analysis estimated the average indirect effect and the proportion of the observed association statistically explained by candidate mediators, including TG, BMI, HDL, FPG, and UA. Given the cross-sectional design, results were interpreted as statistical mediation rather than causal mediation. The proportion statistically explained by each mediator was estimated by comparing models with and without the candidate metabolic factors. Indirect association components were estimated using the product-of-coefficients approach implemented in the mediation package, and the proportion mediated was calculated using log-odds differences between total and direct effects. All analyses were two-sided, with *p* < 0.05 considered statistically significant. Given the large sample size, interpretation emphasized effect size and consistency rather than statistical significance alone. Analyses were hypothesis-driven and involved predefined metabolic and clinical variables, formal correction for multiple testing was not applied. Results were interpreted based on effect estimates, confidence intervals, consistency across analyses, and biological plausibility rather than statistical significance alone.

## 3 Results

### 3.1 Study population and baseline characteristics

A total of 35,468 participants (mean age 46.3 years [SD 11.8]) were included in the analysis. The mean serum 25-hydroxyvitamin D [25(OH)D] concentration was 65.4 nmol/L (SD 26.4). Based on clinical thresholds, 29.59% of participants were vitamin D deficient (<50 nmol/L), 41.90% were insufficient (50–75 nmol/L), and 28.50% had sufficient vitamin D levels (>75 nmol/L). Among the 35,468 participants included in the overall analysis, abdominal ultrasonography data were available for 29,452 individuals. In this ultrasound-assessed subgroup, the prevalence of MASLD was 36.1% (10,639/29,452), with a marked sex disparity (51.1% in males vs. 23.7% in females). Other common ultrasound findings included thyroid nodules (50.5%) and gallbladder polyps (14.3%). Baseline metabolic characteristics were consistent with a metabolically heterogeneous population, with mean BMI of 23.8 kg/m^2^ (SD 3.39), triglycerides 1.45 mmol/L (SD 1.45), and HDL cholesterol 1.45 mmol/L (SD 0.35; Table 1).

**Table 1:** Baseline demographic, metabolic, biochemical, and ultrasonographic characteristics of the study population (*N* = 35,468).

Type	Factors	Number	Mean	SD	Missing rate (%)
Demographic metrics	Age (yrs)	35,468	46.258	11.753	0
Body shape indicators	Height (cm)	33,240	164.473	8.361	6.3
	Weight (Kg)	33,241	64.684	12.799	6.3
	BMI (kg/m^2^)	33,235	23.78	3.393	6.3
	Waistline (cm)	31,444	80.294	10.075	11.3
	Hipline (cm)	31,445	95.107	6.283	11.3
Blood pressure	SBP (mmHg)	4,718	119.57	16.209	86.7
	DBP (mmHg)	4,718	72.958	11.217	86.7
Vitamin D	VitD (nmol/L)	35,467	65.404	26.41	0
Glucose Metabolism	FPG (mmol/L)	35,468	5.019	1.105	0
	HbA1c (%)	35,468	5.715	0.689	0
Blood lipids	TG (mmol/L)	25,499	1.447	1.445	28.1
	TC (mmol/L)	25,499	5.287	1.052	28.1
	HDL (mmol/L)	25,489	1.452	0.348	28.1
	LDL (mmol/L)	25,489	3.18	0.782	28.1
Uric acid	UA (μmol/L)	35,370	345.893	92.416	0.3
Liver function	ALT (U/L)	35,355	23.983	20.56	0.3
	AST (U/L)	35,367	21.954	10.928	0.3
	GGTP (U/L)	17,697	29.333	32.322	50.1
	AKP (U/L)	17,697	68.212	20.703	50.1
Bilirubin	T-Bil (μmol/L)	35,326	14.295	5.493	0.4
	D-Bil (μmol/L)	19,991	2.446	1.057	43.6
	I-Bil (μmol/L)	9,401	11.545	4.451	73.5
Protein	Albumin (g/L)	33,028	44.815	2.681	6.9
	TP (g/L)	33,028	74.689	4.172	6.9
Thyroid function	TSH (mIU/L)	10,803	2.306	3.192	69.5
	FT4 (mIU/L)	10,847	11.229	2.191	69.4
	FT3 (mIU/L)	10,848	5.174	0.944	69.4
Mineral test	Calcium (mmol/L)	189	2.374	0.091	99.5

### 3.2 Correlation of vitamin D with metabolic factors

**Figure 1:**
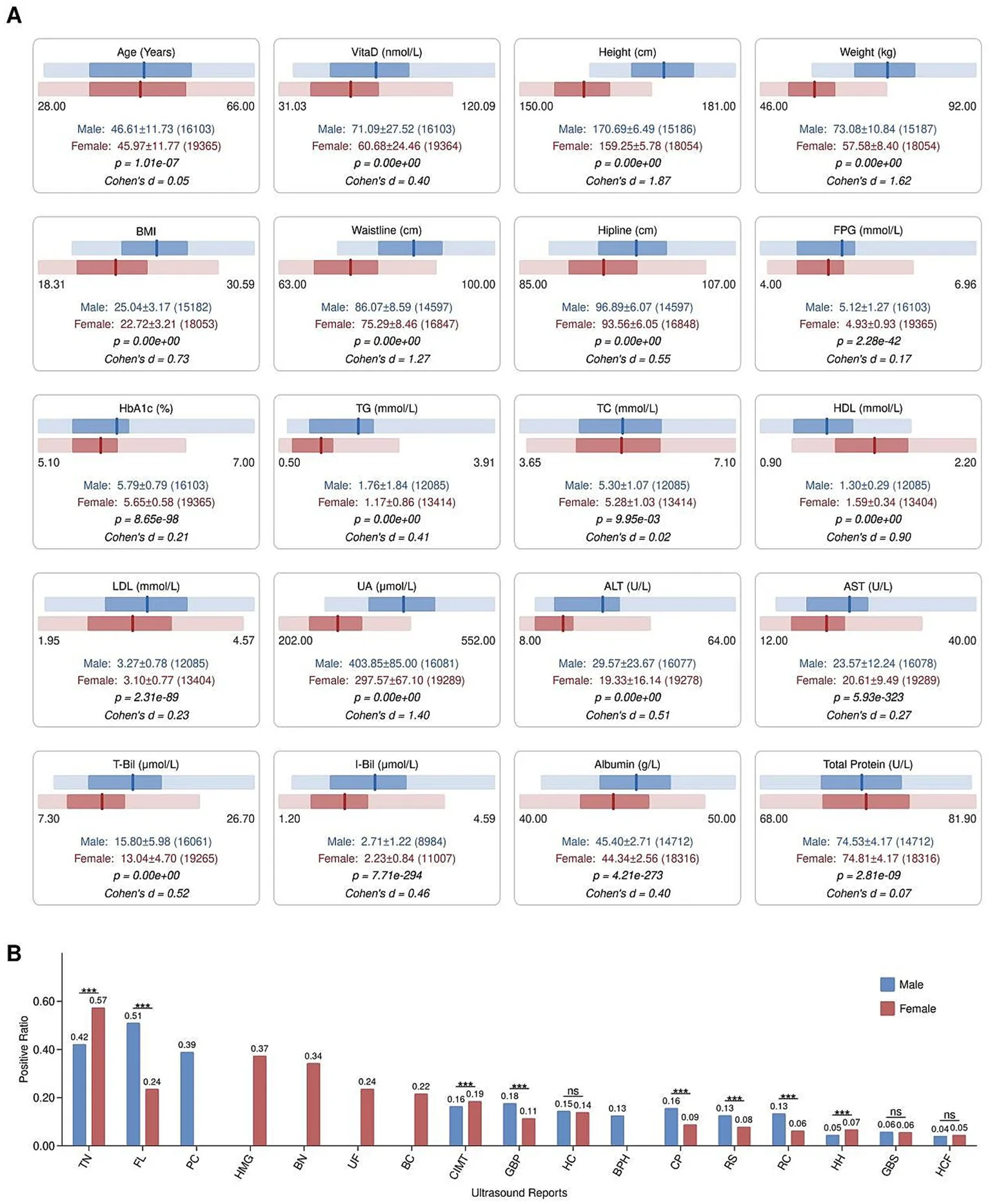
Sex-specific distribution of metabolic parameters and ultrasonographic findings in the study population. **(A)** Comparison of demographic, anthropometric, biochemical, and metabolic variables between male and female participants, including age, serum 25-hydroxyvitamin D [25(OH)D], body composition indices, glycemic markers, lipid profiles, liver function markers, and serum proteins. Data are presented as mean ± standard deviation (SD). Statistical significance between sexes was assessed using two-sided tests, and effect sizes are presented as Cohen’s *d*. **(B)** Sex-specific prevalence of ultrasonographic abnormalities. Blue bars represent males and red bars represent females. TN, thyroid nodules; FL, fatty liver; PC, prostate calcification; HMG, hepatic mass/hemangioma; BN, breast nhodules; UF, uterine fibroids; BC, breast cysts; CMT, carotid mixed plaques/thickening; GBP, gallbladder polyps; HC, hepatic cysts; BPH, benign prostatic hyperplasia; CP, chronic pancreatitis; RS, renal stones; RC, renal cysts; HH, hepatic hemangioma; GBS, gallbladder stones. Statistical significance: **p < 0.001; ns, not significant.

**Figure 2:**
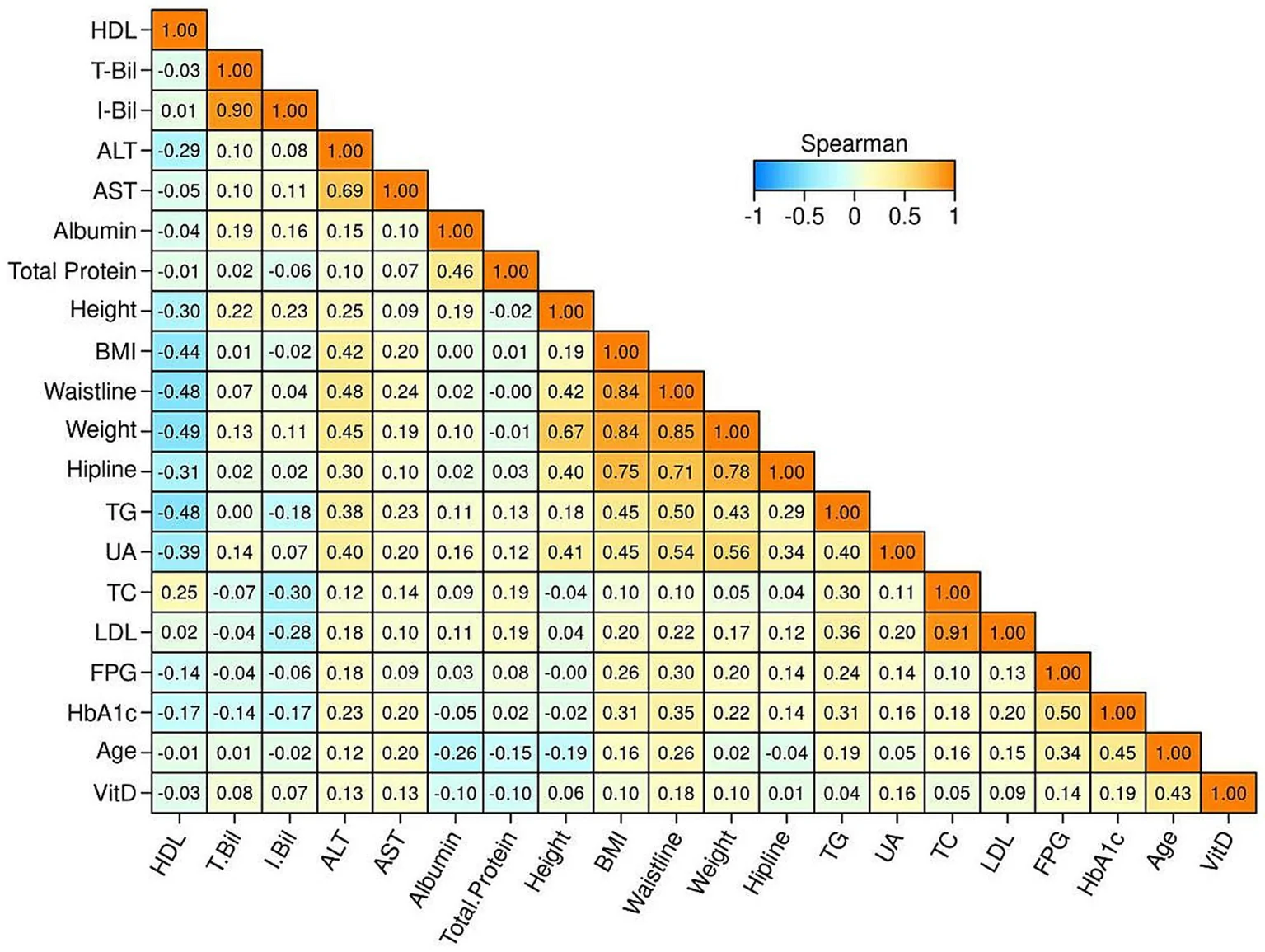
Spearman correlation matrix of serum 25-hydroxyvitamin D [25(OH)D] and metabolic parameters. Heatmap showing pairwise Spearman correlation coefficients (*ρ*) among demographic, anthropometric, biochemical, and metabolic variables. Color intensity represents the strength and direction of the correlations, with blue indicating negative correlations and orange indicating positive correlations. Strong positive correlations were observed among obesity-related parameters, including BMI, waist circumference, and body weight, whereas HDL cholesterol showed inverse associations with multiple metabolic risk markers. Serum 25(OH)D demonstrated its strongest positive correlation with age and relatively weak correlations with glycemic and lipid parameters. BMI, body mass index; TG, triglycerides; TC, total cholesterol; LDL, low-density lipoprotein cholesterol; HDL, high-density lipoprotein cholesterol; FPG, fasting plasma glucose; HbA1c, glycated hemoglobin; UA, uric acid; ALT, alanine aminotransferase; AST, aspartate aminotransferase; T-Bil, total bilirubin; I-Bil, indirect bilirubin; VitD, serum 25-hydroxyvitamin D.

Serum 25-hydroxyvitamin D [25(OH)D] concentrations were higher in males than females (mean 71.1 vs. 60.7 nmol/L; Cohen’s d = 0.40; Figure 1A). Males also had higher triglycerides, uric acid, and liver enzyme levels, whereas HDL cholesterol was higher in females. Large sex differences were observed for anthropometric measures, including height (*d* = 1.87 cm), weight (1.62 kg), and waist circumference (1.27 cm), with moderate differences for BMI (0.73 kg/m^2^), triglycerides (0.41 mmol/L), and HDL cholesterol (*d* = −0.90 mmol/L). Differences in total cholesterol and fasting glucose were minimal despite statistical significance (Figure 1A). Sex disparities were also evident in ultrasound findings (Figure 1B; Supplementary Table S1), with a higher prevalence of fatty liver in males and a higher prevalence of thyroid nodules in females, while other abnormalities showed smaller or non-significant differences. Serum 25-hydroxyvitamin D [25(OH)D] was most strongly correlated with age (years; *ρ* = 0.43), exceeding associations with all other variables (Figure 2). Correlations between vitamin D and metabolic parameters were weak, including HbA1c (%; *ρ* = 0.19), fasting plasma glucose (mmol/L; *ρ* = 0.14), uric acid (μmol/L; *ρ* = 0.16), triglycerides (mmol/L; *ρ* = 0.04), HDL cholesterol (mmol/L; *ρ* = −0.03), and albumin (g/L; *ρ* = −0.10). In contrast, strong intercorrelations were observed among metabolic variables. BMI (kg/m^2^), waist circumference (cm), and weight (kg) were highly correlated (*ρ* = 0.84–0.85) and were positively associated with triglycerides (mmol/L), uric acid (μmol/L), and liver enzymes (U/L), whereas HDL cholesterol (mmol/L) was inversely associated with these parameters (BMI *ρ* = −0.44; triglycerides *ρ* = −0.48). Total cholesterol and LDL cholesterol (both mmol/L) were strongly correlated (*ρ* = 0.91; Figure 2).

### 3.3 Confounding structure and independent associations of vitamin D

**Figure 3:**
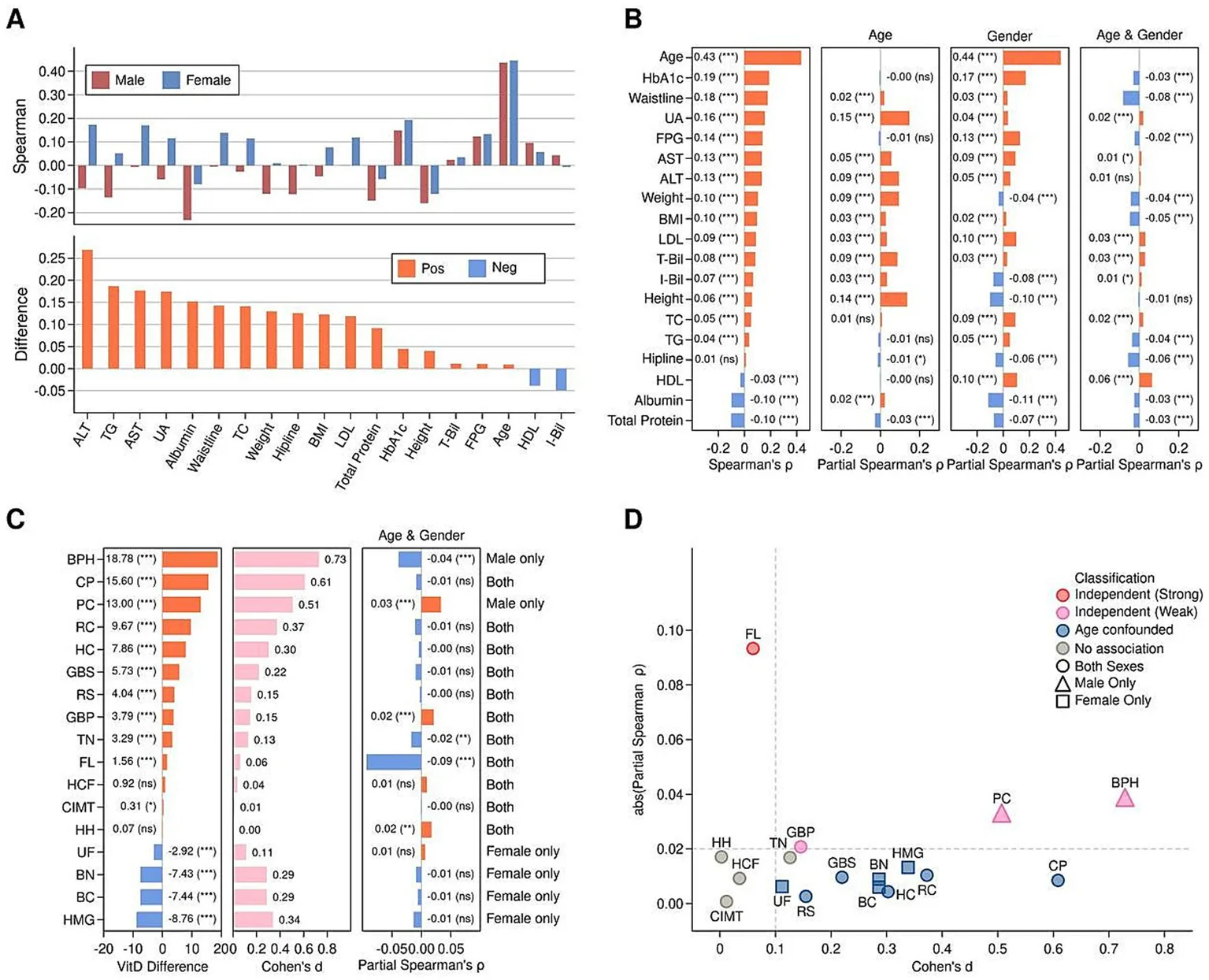
Sex- and age-adjusted associations between serum 25-hydroxyvitamin D [25(OH)D] and metabolic or ultrasonographic parameters. **(A)** Sex-stratified Spearman correlation coefficients between serum 25(OH)D and metabolic variables in males and females (upper panel), together with the absolute differences in correlation strength between positive and negative associations (lower panel). Females generally demonstrated stronger positive correlations between vitamin D and metabolic traits compared with males. **(B)** Crude and partial Spearman correlation analyses of serum 25(OH)D with metabolic variables after adjustment for age, sex, or both age and sex. Adjustment for age markedly attenuated most associations, indicating that age was the principal confounding factor underlying the observed correlations between vitamin D and metabolic traits. Statistical significance is indicated as follows: *p* < 0.05, *p* < 0.01, and *p* < 0.001; ns, not significant. **(C)** Differences in serum 25(OH)D concentrations according to ultrasonographic findings. Left panel shows mean vitamin D differences between participants with and without each ultrasound abnormality; middle panel presents corresponding effect sizes (Cohen’s *d*); right panel shows partial Spearman correlation coefficients adjusted for age and sex. Positive associations with serum 25(OH)D were observed for benign prostatic hyperplasia and prostate calcification, whereas lower vitamin D levels were associated with several female-specific findings, including breast nodules, breast cysts, uterine fibroids, and hepatic masses. **(D)** Integrated classification plot summarizing the relationship between ultrasound abnormalities and serum 25(OH)D based on effect size (Cohen’s *d*) and adjusted association strength (absolute partial Spearman’s *ρ*). Ultrasound findings were categorized as independent strong associations, independent weak associations, age-confounded associations, or no association. Triangle symbols indicate male-specific findings, square symbols indicate female-specific findings, and circles represent abnormalities observed in both sexes. ALT, alanine aminotransferase; AST, aspartate aminotransferase; TG, triglycerides; UA, uric acid; BMI, body mass index; HDL, high-density lipoprotein cholesterol; LDL, low-density lipoprotein cholesterol; HbA1c, glycated hemoglobin; FL, fatty liver; TN, thyroid nodules; PC, prostate calcification; BPH, benign prostatic hyperplasia; CP, chronic pancreatitis; RC, renal cysts; HC, hepatic cysts; GBS, gallbladder stones; RS, renal stones; GBP, gallbladder polyps; HCF, hepatic calcified focus; CIMT, carotid intima-media thickening; HH, hepatic hemangioma; UF, uterine fibroids; BN, breast nodules; BC, breast cysts; HMG, hepatic mass/hemangioma.

Comparison of crude and adjusted analyses revealed a pronounced confounding structure underlying the association between serum 25-hydroxyvitamin D [25(OH)D] and metabolic traits. Several variables that were positively associated with vitamin D in unadjusted analyses including HbA1c, waist circumference, BMI, and body weight underwent direction reversal after adjustment for age and sex, indicating that these associations were largely driven by demographic confounding rather than direct biological relationships (Figures 3A,B). After accounting for these effects, only a limited subset of metabolic variables remained independently associated with vitamin D. Triglycerides showed the strongest inverse association (partial *ρ* ≈ −0.13), followed by adiposity-related measures (*ρ* ≈ −0.06 to −0.07), whereas HDL cholesterol remained positively associated (*ρ* ≈ 0.08). In contrast, associations with total cholesterol, LDL cholesterol, and liver enzymes were fully attenuated, suggesting that their crude relationships with vitamin D were non-independent (Figure 3B). Sex-stratified analyses demonstrated substantial effect heterogeneity. Several metabolic variables, including ALT and triglycerides, exhibited divergent or attenuated associations between males and females, indicating that sex modifies the relationship between vitamin D and metabolic phenotype (Figures 3A,B). A similar pattern was observed for ultrasound-detected abnormalities. Most crude differences in vitamin D levels across clinical conditions were markedly reduced after adjustment, consistent with strong age-related confounding. Notably, fatty liver remained the only condition showing a consistent inverse association with vitamin D after adjustment (partial *ρ* ≈ −0.09), whereas associations with other conditions were negligible (Figures 3C,D). These findings indicate that the apparent metabolic associations of vitamin D are largely attributable to shared demographic and metabolic determinants, with only a limited number of traits demonstrating independent relationships.

### 3.4 Dose–response relationships and mediation of vitamin D–MASLD association

**Figure 4:**
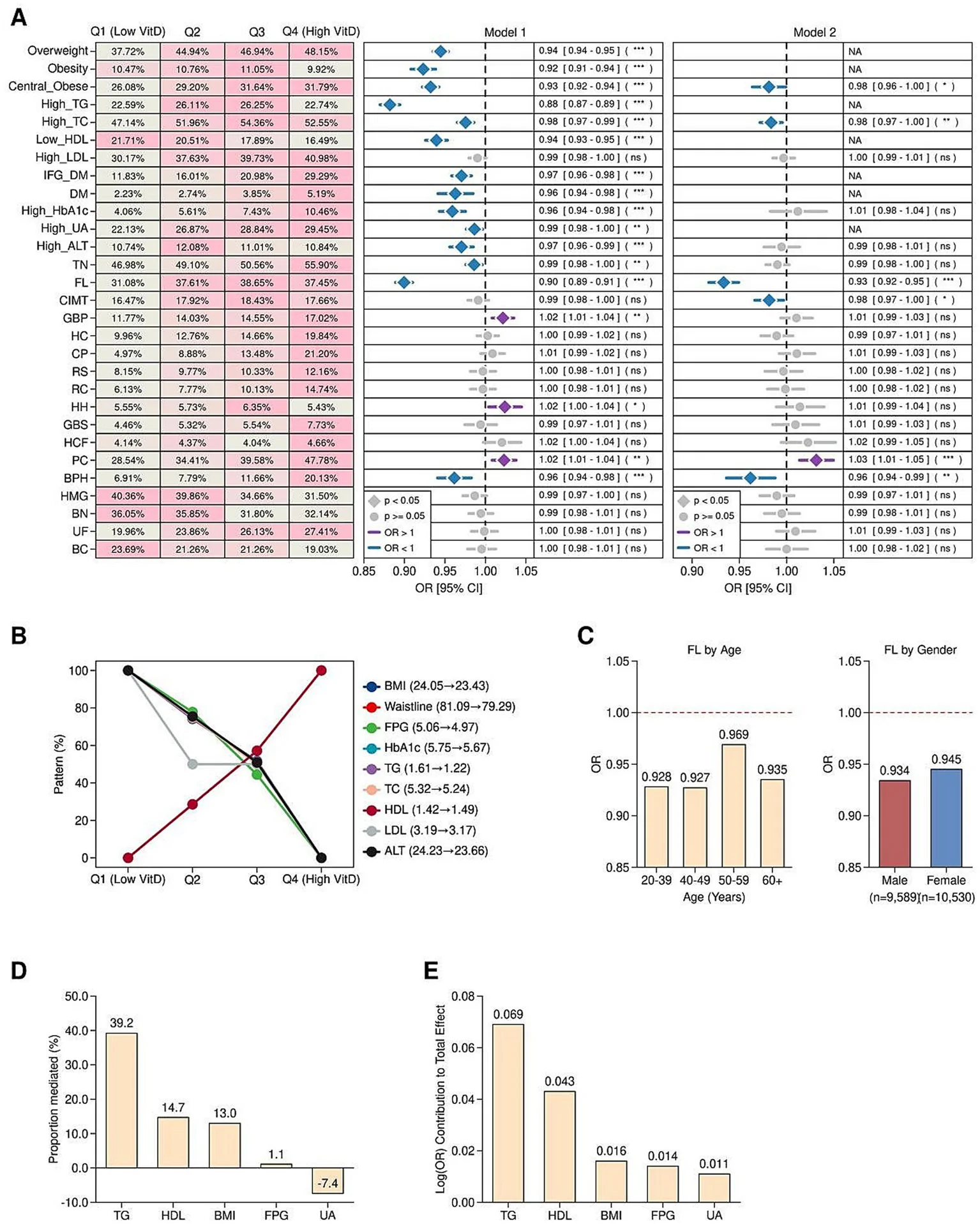
Associations between serum 25-hydroxyvitamin D [25(OH)D] quartiles and metabolic or ultrasonographic phenotypes **(A)** Distribution of metabolic abnormalities and ultrasonographic findings across quartiles of serum 25(OH)D concentration (Q1: Lowest vitamin D; Q4: Highest vitamin D), together with odds ratios (ORs) and 95% confidence intervals (CIs) from logistic regression analyses. Model 1 represents minimally adjusted associations, whereas Model 2 includes multivariable adjustment for major confounding factors, including age and sex where appropriate. Blue markers indicate inverse associations (OR <1), purple markers indicate positive associations (OR >1), and gray markers indicate non-significant findings. Increasing vitamin D levels were associated with lower odds of fatty liver, obesity-related traits, impaired glucose metabolism, and benign prostatic hyperplasia, whereas positive associations were observed for prostate calcification and selected hepatobiliary findings. **(B)** Trend patterns of representative metabolic parameters across vitamin D quartiles. Increasing serum 25(OH)D concentrations were associated with lower BMI, waist circumference, triglycerides, fasting plasma glucose, HbA1c, LDL cholesterol, and alanine aminotransferase levels, while HDL cholesterol increased progressively across quartiles. Values shown in parentheses indicate mean changes from Q1 to Q4. **(C)** Stratified association analysis between serum 25(OH)D and fatty liver (FL) according to age groups and sex. Odds ratios remained below 1.0 across all subgroups, supporting a consistent inverse association between higher vitamin D status and fatty liver prevalence. **(D)** Mediation analysis showing the proportion of the association between serum 25(OH)D and fatty liver explained by metabolic mediators. Triglycerides demonstrated the largest mediating effect, followed by HDL cholesterol and BMI, whereas uric acid showed a small negative mediation effect. **(E)** Relative contribution of individual metabolic factors to the total effect of serum 25(OH)D on fatty liver risk, expressed as log(OR) contribution. Triglycerides and HDL cholesterol accounted for the largest proportions of the total observed association. VitD, serum 25-hydroxyvitamin D; BMI, body mass index; TG, triglycerides; HDL, high-density lipoprotein cholesterol; LDL, low-density lipoprotein cholesterol; FPG, fasting plasma glucose; HbA1c, glycated hemoglobin; UA, uric acid; ALT, alanine aminotransferase; FL, fatty liver; TN, thyroid nodules; CIMT, carotid intima-media thickening; GBP, gallbladder polyps; HC, hepatic cysts; CP, chronic pancreatitis; RS, renal stones; RC, renal cysts; HH, hepatic hemangioma; GBS, gallbladder stones; HCF, hepatic calcified focus; PC, prostate calcification; BPH, benign prostatic hyperplasia; HMG, hepatic mass/hemangioma; BN, breast nodules; UF, uterine fibroids; BC, breast cysts; OR, odds ratio; CI, confidence interval; ns, not significant.

Across quartiles of serum 25-hydroxyvitamin D [25(OH)D], crude prevalence of several metabolic abnormalities increased with higher vitamin D levels (Figure 4A). However, after adjustment for age and sex, higher vitamin D concentrations were associated with more favorable metabolic profiles, including lower triglycerides (1.61 to 1.22 mmol/L), lower body mass index (24.05 to 23.43 kg/m^2^), and higher HDL cholesterol (1.42 to 1.49 mmol/L; Figure 4B). In logistic regression analyses, higher vitamin D levels were associated with lower odds of multiple metabolic abnormalities in models adjusted for age and sex, with the strongest effects observed for hypertriglyceridaemia [OR 0.88 (95% CI 0.87–0.89)] and fatty liver [OR 0.90 (0.89–0.91)] (Figure 4A). After further adjustment for metabolic covariates, most associations were attenuated, whereas the inverse association with fatty liver remained robust [OR 0.93 (0.92–0.95)]. The association between vitamin D and fatty liver was consistent across age groups and sexes, with similar effect sizes observed in all subgroups (age-stratified ORs 0.93–0.97; male 0.93, female 0.95; Figure 4C). Mediation analysis demonstrated that 37.6% of the observed association between vitamin D and fatty liver was statistically explained by metabolic factors, whereas the remaining proportion was not explained by the measured mediators included in the model (Figures 4D,E). Among individual pathways, triglycerides accounted for the largest proportion of the statistically explained association (39.2%), followed by HDL cholesterol (14.7%) and BMI (13.0%), whereas fasting glucose contributed minimally and uric acid showed no significant mediation. These findings indicate that the association between vitamin D and fatty liver is only partially mediated by metabolic risk factors, with a substantial proportion not explained by the measured metabolic factors included in the model.

### 3.5 Determinants of vitamin D and effect modification

**Figure 5:**
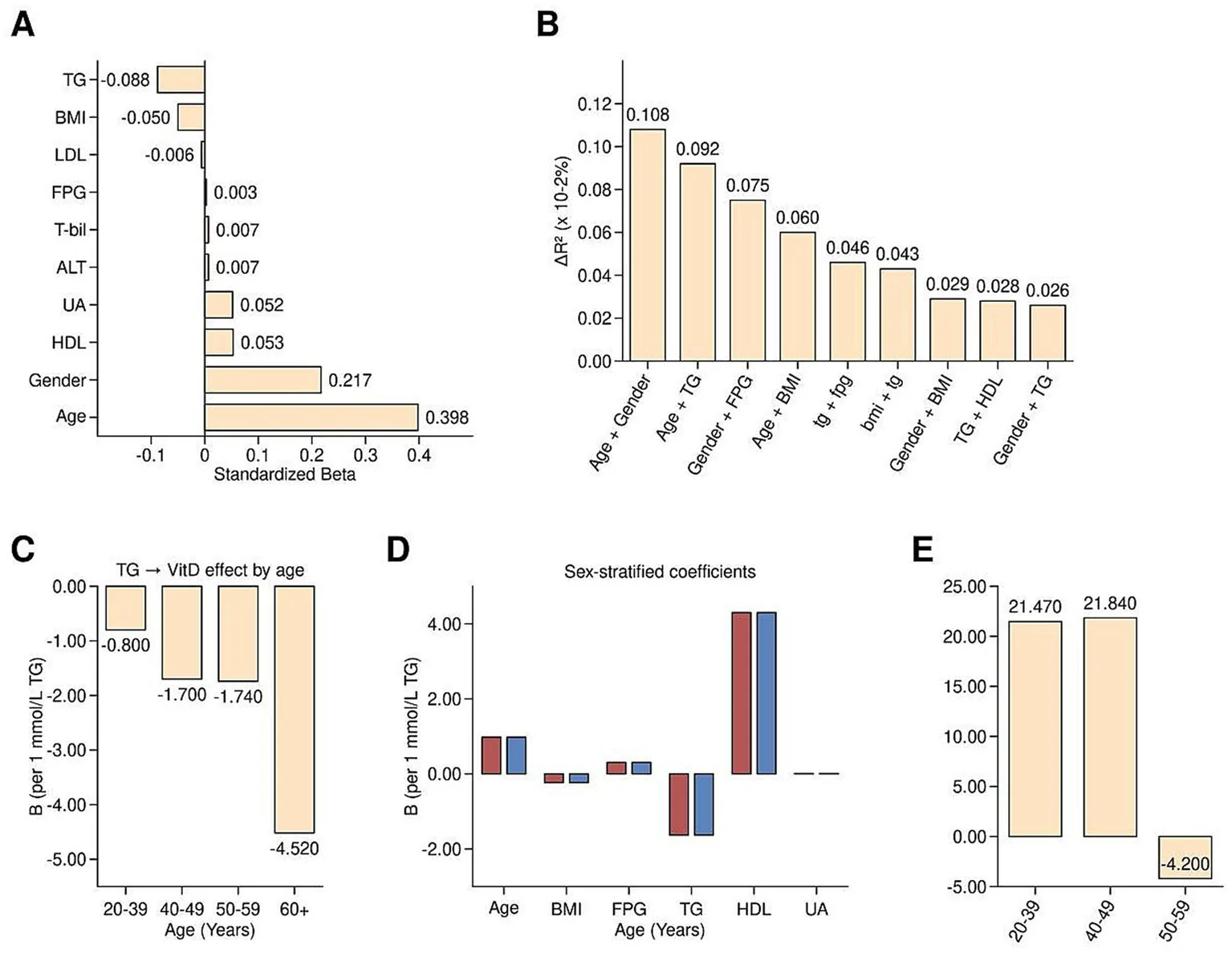
Multivariable determinants and stratified effects of serum 25-hydroxyvitamin D [25(OH)D] associations with metabolic parameters **(A)** Standardized beta coefficients from multivariable regression analysis evaluating independent predictors of serum 25(OH)D concentration. Age and sex demonstrated the strongest positive associations with vitamin D levels, whereas triglycerides (TG) and body mass index (BMI) showed the strongest inverse associations. Smaller positive associations were observed for HDL cholesterol and uric acid. **(B)** Relative contributions of combined metabolic and demographic predictors to the explained variance in serum 25(OH)D levels, expressed as incremental ΔR^2^ values. Models incorporating age, sex, triglycerides, fasting plasma glucose, and BMI contributed most substantially to the variance explained. **(C)** Age-stratified effect estimates for the association between triglycerides and serum 25(OH)D concentration. The inverse relationship between triglycerides and vitamin D became progressively stronger with advancing age, with the largest effect observed in participants aged ≥60 years. **(D)** Sex-stratified regression coefficients demonstrating comparable patterns of association between serum 25(OH)D and metabolic parameters in males and females. Triglycerides consistently showed inverse associations, whereas HDL cholesterol and age showed positive associations across both sexes. **(E)** Age-specific effect estimates illustrating differential associations between serum 25(OH)D and selected metabolic outcomes across age categories. Positive associations were strongest in younger and middle-aged adults, whereas an inverse relationship was observed in participants aged 50–59 years. VitD, serum 25-hydroxyvitamin D; TG, triglycerides; BMI, body mass index; LDL, low-density lipoprotein cholesterol; HDL, high-density lipoprotein cholesterol; FPG, fasting plasma glucose; UA, uric acid; ALT, alanine aminotransferase; T-Bil, total bilirubin; ΔR^2^, incremental explained variance.

In multivariable regression analyses, age (standardized *β* = 0.40) and male sex (*β* = 0.22) were the strongest independent predictors of serum 25-hydroxyvitamin D [25(OH)D] concentrations (Figure 5A; Table 2). Among metabolic variables, triglycerides showed the strongest inverse association (*β* = −0.09), followed by body mass index (*β* = −0.05), whereas HDL cholesterol (*β* = 0.05) and uric acid (*β* = 0.05) were positively associated. Fasting plasma glucose, LDL cholesterol, alanine aminotransferase, and bilirubin showed no independent associations. Incremental model analysis demonstrated that age and sex accounted for the majority of explained variance, with metabolic variables contributing only modest additional explanatory power (Figure 5B; Table 3). The largest incremental contributions were observed for triglycerides and body mass index. Marked effect modification by age was observed for the association between triglycerides and vitamin D. The magnitude of the inverse association increased progressively across age groups, from B = −0.80 in younger adults to B = −4.52 in those aged ≥60 years (Figure 5C). Sex-stratified analyses demonstrated consistent patterns across males and females, although the magnitude of associations differed, with stronger effects of adiposity and triglycerides observed in females (Figure 5D). In models incorporating clinical outcomes, individuals with fatty liver had lower vitamin D concentrations (−4.2 nmol/L) independent of metabolic covariates, indicating an association beyond traditional metabolic pathways (Figure 5E). Together, these findings indicate that vitamin D levels are primarily determined by demographic factors, with metabolic influences exerting secondary and context-dependent effects.

**Table 2:** Multivariable regression model of independent predictors of serum 25-hydroxyvitamin D [25(OH)D] concentrations with clinical interpretation.

Predictor	B (unstandardized)	95% CI	*β* (standardized)	*p*-value	Clinical interpretation
Age (per year)	0.907	[+0.876, +0.937]	0.398	< 10^−300^ ***	Each additional year → VitD +0.91 nmol/L
Gender (Male)	11.42	[+10.63, +12.22]	0.217	6 × 10^−174^ ***	Males 11.4 nmol/L higher than females
TG (per mmol/L)	−1.725	[−2.12, −1.33]	−0.088	1.9 × 10^−17^ ***	Strongest negative metabolic predictor
HDL (per mmol/L)	3.975	[+2.74, +5.21]	0.053	3.2 × 10^−10^ ***	Higher HDL → higher VitD
UA (per μmol/L)	0.015	[+0.010, +0.019]	0.052	5.9 × 10^−11^ ***	+100 μmol/L → VitD +1.4 nmol/L
BMI (per kg/m^2^)	−0.383	[−0.49, −0.28]	−0.05	1.2 × 10^−12^ ***	Each +1 kg/m^2^ → VitD -0.38
FPG (per mmol/L)	0.062	[−0.27, +0.39]	0.003	0.712 ns	Not independently associated
LDL (per mmol/L)	−0.215	[−0.66, +0.23]	−0.006	0.339 ns	Not independently associated
ALT (per U/L)	0.009	[−0.006, +0.024]	0.007	0.261 ns	Not independently associated
Total bilirubin	0.035	[−0.022, +0.091]	0.007	0.229 ns	Not independently associated

***p < 0.001.

**Table 3:** Comparison of multivariable regression models evaluating determinants of serum 25-hydroxyvitamin D [25(OH)D] concentrations.

Model	*R* ^2^	*n*	*k*	Key finding
M1: Age + sex	0.195	35,467	2	Baseline: demographics explain 19.5%
M4: Full metabolic	0.206	23,701	10	6 significant: age, sex, TG, HDL, UA, BMI; 4 ns: FPG, LDL, ALT, TBil
M5: + MASLD	0.218	20,059	11	MASLD: B = −4.2, *p* = 2.2 × 10^−21^ (independent of all metabolic covariates)
M6: TyG index	0.21	23,701	9	TyG per +1 unit → VitD −5.72 (insulin resistance proxy)
M7: Interactions	0.001	23,772	1	8/9 significant; age×sex strongest (ΔR^2^ = 0.11%)
M8: Sex-stratified	♂0.178 / ♀0.167	11,340/12,361	8	FPG: ♂ ns / ♀ significant negative; BMI: ♀ effect 2 × stronger
M9: Age-stratified TG	0.048–0.092	2,791–8,419	5	TG B: −0.80 (age 20s) → −4.52 (age 60+), 5.6-fold gradient

## 4 Discussion

In this large retrospective health-screening cohort study, we demonstrate that circulating 25-hydroxyvitamin D [25(OH)D] is inversely associated with MASLD, and that this relationship persists after comprehensive adjustment for metabolic risk factors, suggesting an association that remained after adjustment for measured metabolic factors. By integrating correlation structure, multivariable modeling, and mediation analysis, our findings provide a refined framework for understanding the role of vitamin D in hepatic steatosis, distinguishing between confounded associations and associations statistically related to measured metabolic factors. MASLD is currently the most prevalent chronic liver disease worldwide, affecting approximately 30–32% of the global adult population, with an even higher (40%) burden reported in Asian populations (1, 19, 20). This rising prevalence is closely linked to the global epidemic of obesity and metabolic syndrome, yet MASLD is increasingly recognized in non-obese individuals, highlighting the need to identify additional modifiable risk factors (21). Vitamin D deficiency has emerged as a potential contributor, with accumulating epidemiological and mechanistic evidence suggesting its involvement in hepatic lipid metabolism and inflammation (22). However, previous studies have been limited by inconsistent findings, largely due to inadequate control of confounding metabolic factors.

A central observation of this study is the dominant influence of demographic factors particularly age and gender on circulating vitamin D levels. Age showed the strongest correlation with 25(OH)D, and together with sex accounted for the majority of explained variance. This finding is consistent with prior epidemiological studies indicating that vitamin D levels are strongly shaped by behavioral and environmental factors, including supplementation, healthcare access, and lifestyle patterns. The magnitude of this effect underscores a critical methodological issue in vitamin D research: failure to adequately account for demographic structure can substantially distort associations with metabolic outcomes. Indeed, a key strength of our analysis lies in the explicit characterization of confounding. Several metabolic variables, including BMI, waist circumference, and HbA1c, showed positive associations with vitamin D in crude analyses but reversed direction after adjustment. This pattern is indicative of strong confounding and is consistent with previous observations of paradoxical associations in nutritional epidemiology. Prior studies reporting inconsistent or even positive associations between vitamin D and adverse metabolic profiles may therefore reflect residual confounding rather than true biological effects. By systematically comparing crude and adjusted correlations, our findings demonstrate that many previously reported associations are non-independent. After accounting for confounding, only a limited subset of metabolic factors remained independently associated with vitamin D. Triglycerides emerged as the strongest inverse correlate, followed by adiposity-related measures, while HDL cholesterol showed a modest positive association. In contrast, total cholesterol, LDL cholesterol, and liver enzymes were no longer independently associated. These findings align with recent meta-analyses demonstrating that vitamin D supplementation is associated with improvements in triglycerides and HDL cholesterol, but not consistently with LDL or total cholesterol (23). Similarly, mechanistic studies indicate that vitamin D influences lipid metabolism through vitamin D receptor (VDR)-mediated suppression of hepatic lipogenesis and modulation of adipocyte function (24). The absence of independent associations with glucose-related parameters further suggests that vitamin D may exert more pronounced effects on lipid pathways than on glycaemic regulation.

Importantly, MASLD emerged as the only clinical outcome that maintained a consistent inverse association with vitamin D after full multivariable adjustment. This finding is highly relevant given the global burden of MASLD and its role as a major driver of liver-related and cardiometabolic morbidity. Previous observational studies and meta-analyses have consistently reported an inverse association between vitamin D levels and MASLD prevalence (25). However, these studies have often been limited by incomplete adjustment for confounders. Our results extend this data by demonstrating that the association persists even after controlling for key metabolic mediators, including BMI, triglycerides, HDL, glucose, and uric acid, thereby providing evidence that the association persists after adjustment for measured metabolic factors. The dose–response analysis further supports this interpretation. While crude prevalence of metabolic abnormalities appeared higher in individuals with higher vitamin D levels, these patterns were fully explained by age differences across vitamin D strata. After adjustment, higher vitamin D concentrations were associated with more favorable metabolic profiles, including lower triglycerides and BMI and higher HDL cholesterol. This finding highlights the critical importance of distinguishing between crude and adjusted associations, particularly in large observational datasets where confounding by age and sex can be substantial, a phenomenon widely recognized in epidemiological analyses of nutritional exposures and cardiometabolic outcomes (11). However, the possibility of reverse causation should be considered when interpreting the association between vitamin D status and MASLD. Because this study was cross-sectional, it remains unclear whether reduced 25(OH)D concentrations contribute to MASLD development or represent a consequence of metabolic and hepatic dysfunction. MASLD may itself influence vitamin D homeostasis through impaired hepatic metabolism of vitamin D, altered hydroxylation processes, increased adipose tissue sequestration of vitamin D, and inflammation-associated changes in vitamin D signaling. Therefore, the observed inverse association may reflect a complex bidirectional relationship between vitamin D status and hepatic steatosis rather than a direct causal effect. Future prospective studies with repeated measurements of vitamin D status and liver outcomes are needed to establish temporal relationships and clarify causality. Although observational studies have consistently reported associations between lower vitamin D status and adverse metabolic profiles, evidence from randomized controlled trials evaluating vitamin D supplementation remains heterogeneous. Recent meta-analyses of randomized trials suggest that vitamin D supplementation may improve selected metabolic parameters, including lipid profiles and glycemic-related outcomes, in specific populations; however, the magnitude of these effects varies according to baseline vitamin D status, obesity status, supplementation dose, and intervention duration (26, 27). Therefore, current evidence does not support vitamin D supplementation as an established therapeutic intervention for MASLD, and further well-designed clinical trials are required to determine whether vitamin D correction can modify hepatic steatosis or disease progression. In the context of MASLD, nutritional interventions such as vitamin E supplementation have demonstrated potential benefits for liver enzymes, steatosis, and fibrosis-related outcomes in selected patients, but their clinical applicability remains under investigation (28). Therefore, prospective clinical trials are required to determine whether correction of vitamin D deficiency has meaningful effects on MASLD development or progression. Mediation analysis provides additional insight into the pathways linking vitamin D to MASLD. We found that approximately 38% of the association was mediated through metabolic factors, primarily triglycerides, HDL cholesterol, and BMI, while the remaining proportion was not explained by the measured metabolic factors included in the model. The predominance of triglycerides as a mediator is consistent with both epidemiological and experimental evidence linking vitamin D to hepatic lipid metabolism. In contrast, the minimal contribution of fasting glucose suggests that glycaemic pathways play a limited role in this relationship. These findings suggest that the observed association between vitamin D and MASLD may involve both metabolic factors and additional factors not captured in the present analysis (13, 24). The remaining unexplained association may reflect unmeasured biological, environmental, or metabolic factors; however, this cannot be interpreted as evidence of a causal biological effect. Experimental studies have shown that vitamin D signaling through the VDR modulates hepatic lipid accumulation, inflammation, and fibrogenesis. Vitamin D has been reported to suppress pro-inflammatory cytokine pathways and reduce hepatic stellate cell activation, thereby attenuating fibrosis progression (29). Additionally, vitamin D deficiency has been linked to adipose tissue dysfunction and chronic low-grade inflammation, both of which contribute to hepatic steatosis (24). These mechanisms provide a plausible biological basis for the independent association observed in our study.

Despite strong observational associations, interventional studies have yielded mixed results. Recent meta-analyses of vitamin D supplementation in MASLD have demonstrated modest improvements in lipid parameters and liver enzymes but inconsistent effects on hepatic steatosis and clinical outcomes (30). This discrepancy may reflect heterogeneity in study populations, baseline vitamin D status, and duration of intervention, as well as the possibility that vitamin D exerts preventive rather than therapeutic effects. Our findings demonstrate a persistent association between vitamin D status and MASLD after adjustment for measured metabolic factors. Whether this association reflects a causal relationship or reverse causation requires further investigation. Given the high prevalence of both vitamin D insufficiency and MASLD, population-level screening and nutritional interventions may represent practical strategies for early metabolic disease prevention. In particular, targeted vitamin D assessment in individuals with metabolic risk factors may help identify high-risk populations for early intervention. Another important finding is the presence of effect modification by age and sex. The strength of the association between triglycerides and vitamin D increased with age, suggesting that lipid-mediated pathways may become more prominent in older individuals (31). Sex-stratified analyses revealed differences in the magnitude of associations for adiposity and lipid parameters, which may reflect underlying differences in fat distribution, hormonal regulation, and vitamin D metabolism (32). These findings are consistent with previous studies demonstrating sex-specific metabolic effects of vitamin D and highlight the importance of stratified analyses in future research. Future studies should evaluate individualized vitamin D optimization strategies based on metabolic phenotype, sex, and age, which may improve the precision and effectiveness of preventive interventions for metabolic liver disease. The determinants of vitamin D levels indicate that demographic factors remain the primary drivers of circulating 25(OH)D, with metabolic variables contributing only modestly to overall variance. This observation underscores the importance of environmental and behavioral determinants such as sunlight exposure, diet, and supplementation which are known to play dominant roles in vitamin D status globally. It also highlights that metabolic associations with vitamin D should be interpreted within the broader context of these upstream determinants.

Despite the strengths of the large sample size and comprehensive analytical approach, several considerations should be noted. The present cross-sectional design precludes causal inference. However, the consistency of associations across multiple analytical approaches, including mediation and stratified analyses, supports the robustness of the observed relationships. MASLD diagnosis was based on ultrasonography rather than histological assessment. Although ultrasonography is widely used for large-scale epidemiological studies, information regarding ultrasound equipment, radiologist blinding, and interobserver agreement was unavailable, which may introduce some degree of misclassification. Important determinants of vitamin D status, including sunlight exposure, dietary intake, and vitamin D supplementation, were not available in the present dataset. In addition, other lifestyle-related and socioeconomic factors, including alcohol consumption, smoking status, physical activity, seasonal variation, occupation, income, and education level, could not be assessed or adjusted for. These factors may influence both circulating 25(OH)D concentrations and metabolic outcomes and may contribute to residual confounding. Although we adjusted for major demographic and metabolic factors and observed consistent associations across multiple analyses, the possibility of residual confounding cannot be excluded. Future prospective studies incorporating comprehensive lifestyle, environmental, and socioeconomic information are needed to further clarify the relationship between vitamin D status and MASLD. In addition, missing data arising from routine health-screening practices may have introduced selection bias, particularly for variables that were not measured in all participants. Although variables with substantial missingness were excluded, the potential impact of incomplete data on the observed associations cannot be completely excluded. Although the study population was derived from a large Chinese cohort, the biological mechanisms identified are consistent with global evidence, supporting the broader relevance of these findings.

## 5 Conclusion

In this large retrospective health-screening cohort study, Vitamin D status was independently associated with lower odds of MASLD beyond traditional metabolic factors. While part of this association was statistically explained by lipid metabolism and adiposity-related factors, a substantial proportion was not explained by the measured mediators included in the model, suggesting that additional unmeasured factors may contribute to the observed association. These findings provide new insight into the complex interplay between vitamin D and metabolic health and highlight its potential relevance as an associated marker of metabolic liver health. Given the high prevalence of vitamin D insufficiency and MASLD, optimizing vitamin D status warrants further investigation as a potential component of metabolic health strategies for metabolic disease prevention. Future prospective and interventional studies are needed to validate these associations and clarify their clinical implications.

## Data Availability

The original contributions presented in the study are included in the article/Supplementary material, further inquiries can be directed to the corresponding author/s.
