# Supplementary material for: Vitamin D status, metabolic determinants, and association with MASLD: retrospective health-screening cohort mediation analysis
Source: Front Nutr. 2026 Sep 3;13:1922441. doi: 10.3389/fnut.2026.1922441 (PMC13582490; doi:10.3389/fnut.2026.1922441)
Supplement: Supplementary file 1 [file Table_1.docx]

**Supplementary Table S1: Prevalence of ultrasonographic abnormalities in the study population according to sex**

| **Type** | **Factors** | **Positive** | **Total** | **Total Positive Rate** | **Male Positive** | **Male Positive Rate** | **Female Positive** | **Female Positive Rate** |
| --- | --- | --- | --- | --- | --- | --- | --- | --- |
| Demographic Metrics | Gender | NA | 35468 | NA | 19365 | NA | 16103 | NA |
| Ultrasound Reports | FL | 10639 | 29452 | 36.1 | 6833 | 51.1 | 3806 | 23.7 |
|  | HC | 4181 | 29452 | 14.2 | 1939 | 14.5 | 2242 | 13.9 |
|  | HH | 1698 | 29452 | 5.8 | 604 | 4.5 | 1094 | 6.8 |
|  | HCF | 1266 | 29452 | 4.3 | 541 | 4 | 725 | 4.5 |
|  | GBP | 4207 | 29452 | 14.3 | 2367 | 17.7 | 1840 | 11.4 |
|  | GBS | 1686 | 29452 | 5.7 | 780 | 5.8 | 906 | 5.6 |
|  | TN | 14885 | 29452 | 50.5 | 5642 | 42.2 | 9243 | 57.5 |
|  | HT | 144 | 29452 | 0.5 | 36 | 0.3 | 108 | 0.7 |
|  | HMG | 6013 | 29452 | 20.4 | 0 | 0 | 6013 | 37.4 |
|  | BN | 5526 | 29452 | 18.8 | 0 | 0 | 5526 | 34.4 |
|  | BC | 3486 | 29452 | 11.8 | 0 | 0 | 3486 | 21.7 |
|  | UF | 3813 | 29452 | 12.9 | 0 | 0 | 3813 | 23.7 |
|  | OC | 96 | 29452 | 0.3 | 0 | 0 | 96 | 0.6 |
|  | RC | 2825 | 29452 | 9.6 | 1803 | 13.5 | 1022 | 6.4 |
|  | RS | 2963 | 29452 | 10.1 | 1698 | 12.7 | 1265 | 7.9 |
|  | BPH | 1682 | 29452 | 5.7 | 1682 | 12.6 | 0 | 0 |
|  | PC | 5214 | 29452 | 17.7 | 5214 | 39 | 0 | 0 |
|  | CP | 3519 | 29452 | 11.9 | 2096 | 15.7 | 1423 | 8.8 |
|  | CIMT | 5184 | 29452 | 17.6 | 2194 | 16.4 | 2990 | 18.6 |

*Note: SBP: Systolic pressure; DBP: Diastolic pressure; VitD: Vitamin-D; FPG: Fasting Plasma Glucose; TG: Triglyceride; TC: Total Cholesterol; HDL:High-Density Lipoprotein; LDL: Low-Density Lipoprotein; UA: Urea Acid; ALT: Alanine Aminotransferase; AST: Aspartate Aminotransferase; GGTP: Gamma-Glutamyl Transferase; AKP: Alkaline Phosphatase; T0Bil: Total Bilirubin; D-Bil: Direct Bilirubin; I-Bil: Indirect Bilirubin; Albumin: Albumin; TP: Total Protein; TSH: Thyroid-Stimulating Hormone; FT4: Free Thyroxine; FT3: Free: riiodothyronine; Calcium: Serum Calcium; FL: Fatty Liver; HC: Hepatic Cyst; HH: Hepatic Hemangioma; HCF:	Hepatic Calcification Focus; GBP:	Gallbladder Polyp; GBS:	Gallbladder Stone; TN: Thyroid Nodule; HT:	Hashimoto's Thyroiditis; HMG:	Hyperplasia of Mammary Glands; BN:	Breast Nodule; BC:	Breast Cyst; UF: Uterine Fibroid; OC: Ovarian Cyst; RC:Renal Cyst;;RS:	Renal Stone;BPH:	Benign Prostatic Hyperplasia;PC:	Prostatic Calcification; CP:	Carotid Plaque; CIMT:	Carotid Intima-Media Thickness*
